# Reversal of glucose intolerance in rat offspring exposed to ethanol before birth through reduction of nuclear skeletal muscle HDAC expression by the bile acid TUDCA

**DOI:** 10.14814/phy2.12195

**Published:** 2014-12-23

**Authors:** Xing‐Hai Yao, Khanh H. Nguyen, B. L. Grégoire Nyomba

**Affiliations:** 1 Department of Internal Medicine, University of Manitoba, Winnipeg, Manitoba, Canada

**Keywords:** Endoplasmic reticulum, histone deacetylase, insulin resistance, prenatal alcohol exposure

## Abstract

Prenatal ethanol exposure causes cellular stress, insulin resistance, and glucose intolerance in adult offspring, with increased gluconeogenesis and reduced muscle glucose transporter‐4 (glut4) expression. Impaired insulin activation of Akt and nuclear translocation of histone deacetylases (HDACs) in the liver partly explain increased gluconeogenesis. The mechanism for the reduced glut4 is unknown. Pregnant rats were gavaged with ethanol over the last week of gestation and adult female offspring were studied. Some ethanol exposed offspring was treated with tauroursodeoxycholic acid (TUDCA) for 3 weeks. All these rats underwent intraperitoneal glucose tolerance and insulin tolerance tests. The expression of glut4, HDACs, and markers of endoplasmic reticulum (ER) unfolded protein response (XBP1, CHOP, ATF6) was examined in the gastrocnemius muscle fractions, and in C2C12 muscle cells cultured with ethanol, TUDCA, and HDAC inhibitors. Non‐TUDCA‐treated rats exposed to prenatal ethanol were insulin resistant and glucose intolerant with reduced muscle glut4 expression, increased ER marker expression, and increased nuclear HDACs, whereas TUDCA‐treated rats had normal insulin sensitivity and glucose tolerance with normal glut4 expression, ER marker expression, and HDAC levels. In C2C12 cells, ethanol reduced glut4 expression, but increased ER makers. While TUDCA restored glut4 and ER markers to control levels and HDAC inhibition rescued glut4 expression, HDAC inhibition had no effect on ER markers. The increase in nuclear HDAC levels consequent to prenatal ethanol exposure reduces glut4 expression in adult rat offspring, and this HDAC effect is independent of ER unfolded protein response. HDAC inhibition by TUDCA restores glut4 expression, with improvement in insulin sensitivity and glucose tolerance.

## Abstract (precis)

Alcohol consumption during pregnancy increases nuclear expression of histone deacetylases and endoplasmic response in skeletal muscle, which reduce glucose transporter 4 and in part alter glucose tolerance in offspring. These anomalies are reversed by treatment with tauroursodeoxycholic acid.

## Introduction

Prenatal ethanol exposure is well known to cause fetal alcohol spectrum disorders, which, among others, result in cognitive and behavioral alterations due to defective central nervous system development in offspring. Studies in human offspring have suggested that prenatal ethanol exposure also results in glucose intolerance (Castells et al. 1981) and obesity especially in females (Werts et al. 2014). Animal studies have demonstrated increased adiposity, in vivo insulin resistance, and glucose intolerance in adult offspring of both sexes (Chen and Nyomba 2003a,b; Yao and Nyomba 2007; Yao et al. 2013; Harper et al. 2014), occurring after ethanol exposure as short as 7 days regardless of gestational age (Yao et al. 2013), and even transmitted to the next generation (Harper et al. 2014). In a recent study, we have found that exposure of rat dams to alcohol for only 1 week at the end of pregnancy leads to glucose intolerance in adult male offspring (Yao et al. 2013). In skeletal muscle, prenatal ethanol‐induced insulin resistance is characterized by a reduction of glucose transporter (glut)‐4 expression (Chen and Nyomba 2003a,b) and impaired insulin signaling in the phosphoinositide 3‐kinase pathway (Elton et al. 2002; Chen et al. 2005; Yao and Nyomba 2007). Prenatal ethanol also increases hepatic and skeletal muscle expression of tribbles‐3 and PTEN (phosphatase and tensin homolog deleted on chromosome 10), which are markers of cellular stress and can inhibit Akt activation (Yao and Nyomba 2007, 2008). However, the mechanism for reduced glut4 expression associated with prenatal ethanol exposure remains unknown.

Recent studies have suggested that the acetylation status of core histones near the glut4 promoter may affect glut4 expression (Takigawa‐Imamura et al. 2003; Raychaudhuri et al. 2008; Raichur et al. 2012). Protein acetylation status is kept in balance, among others, through opposing actions of histone acetylase (HAT) and histone deacetylase (HDAC) enzymes and plays important functions in gene expression by regulating transcription factor access to chromatin (Narlikar et al. 2002). Class I HDACs (e.g., HDAC1 and 3) primarily localize to the nucleus, whereas class II HDACs (e.g., HDAC4, 5, 7, 9) have a primarily cytoplasmic localization and translocate to and from the nucleus in response to cellular cues (McKinsey et al. 2001). Cellular stress may result in nuclear localization of class II HDACs which, by forming complexes with class I HDACs, inhibit gene expression (Miura et al. 2008; Agudelo et al. 2011; Kahali et al. 2012; Liu et al. 2012; Zhang et al. 2014). It has been suggested that class I HDACs may be involved in the negative transcriptional regulation of endoplasmic reticulum (ER) chaperones (Baumeister et al. 2005). It has also been shown that deacetylation by HDACs can target nonhistone proteins in the ER, mitochondria, and cytoplasm, and that certain HDACs are upregulated by ER stress (Kahali et al. 2012). For example, deacetylation of Akt inhibitors PTEN and tribbles‐3 by HDACs results in insulin resistance due to increased Akt inhibition by these proteins (Yao and Nyomba 2008). Some HDACs have also been shown to impair glucose metabolism by inhibiting glut4 expression through deacetylation of core histones (Takigawa‐Imamura et al. 2003; Raychaudhuri et al. 2008; Raichur et al. 2012). We have previously shown that HDAC levels are increased in the liver nucleus of rats exposed to ethanol before birth in association with increased gluconeogenic genes, and that these anomalies are reversed by the bile acid TUDCA, which also modulates ER function (Yao et al. 2013). In the present study, we hypothesized that the reduced glut4 expression in rats prenatally exposed to ethanol can in part be explained by HDAC levels.

## Materials and Methods

### Chemicals and antibodies

N‐(4‐chloro‐3‐trifluoromethyl‐phenyl)‐2‐ethoxy‐6‐pentadecyl‐benzamide (CTPB, sc‐202558), garcinol (sc‐200891), p300 siRNA (m, sc‐29432), control siRNA‐A (sc‐37007), siRNA dilute RNAse‐free H_2_O (Lot#L2012), siRNA dilute RNAse‐free H_2_O (Lot#A1713), siRNA transfection medium (sc‐36868), siRNA transfection reagent (sc‐29528), siRNA dilute buffer (sc‐29527), and antibodies against glut4 (C20, sc‐1608), HDAC1 (H51, sc‐7872), HDAC3 (H99, sc‐11417), HDAC4 (H‐92, sc‐11418), HDAC5 (G‐18, sc‐5250), HDAC7 (N‐18, sc‐11489), HDAC9 (H45, sc‐28732), PTEN (A2B1, sc‐7974), tribbles‐3 (H19, sc‐34211), and *β*‐actin (I‐19, sc‐1616), as well as goat anti‐rabbit IgG‐horseradish peroxidase (HRP, sc‐2004) and donkey anti‐mouse IgG‐HRP (sc‐2314) were purchased from Santa Cruz Biotechnology (Santa Cruz, CA). Akt and phospho‐Akt Bio‐Plex Pro Assay kits were from Bio‐Rad (Hercules, CA). Resveratrol (#R5010), suberoylanilide hydroxamic acid (SAHA, #SML0061), tauroursodeoxycholic acid sodium salt (TUDCA, #T0266) and all other chemicals were from Sigma–Aldrich (Oakville, ON, Canada).

### Ethics statement

All studies were approved by the Committee for Animal Use in Research and Teaching of the University of Manitoba prior to commencement of the studies, in full compliance with the Canadian Council on Animal Care who has certified that the animal care and use program at the University of Manitoba is in accordance with the standards of Good Animal Practice.

### Animals and experimental design

Animal experiments were performed as described (Chen and Nyomba 2003a,b; Dembele et al. 2006; Yao and Nyomba 2007, 2008). Briefly, virgin Sprague–Dawley rats from Charles River Canada (Saint Constant, QC) were housed individually and given free access to normal chow. The rats were randomly divided into four groups (*n* = 6–7/group) and pregnancy was timed by the vaginal plug method. Two groups were given ethanol (2 g/kg, 36%) by gavage twice daily at 9:00 AM and 4:00 PM from pregnancy day 15 to term, and the other two groups were given the same volume of water instead of ethanol. This amount of ethanol is moderate in rats (Keshavarzian et al. 2001) and results in alcoholemia of 115 mg/dL 2 h after ingestion, which is similar to levels found in nonintoxicated human alcoholics (Urso et al. 1981), and decreases to 70 mg/dL at 4 h, which is considered safe for driving (Chen and Nyomba 2003a). A recent study has confirmed our previous observations that, with this amount of maternal alcohol in rodents, there are no visible birth defects and no abnormal number of pups, difference in sex ratio, or miscarriages (Zhou et al. 2012). One group of the nonethanol groups was pair‐fed the amount of chow consumed by the ethanol group, whereas the other group (control) was given free access to chow. Pair‐feeding, matching for caloric intake, is a common practice in these experiments because of data showing slight quantitative reduction of food intake in rats consuming ethanol (Chen and Nyomba 2003a,b). Offspring body weight was recorded daily from day 1–7 and then weekly until 16 weeks of age. After weaning onto normal chow, female offspring from one of the ethanol groups of dams were given daily intraperitoneal injections of TUDCA (15 mg/kg) for 3 weeks starting at 13 weeks of age (ethanol‐TUDCA), whereas the others were given normal saline. The TUDCA dose and time of administration were previously reported in this rat model (Yao et al. 2013) and in insulin‐resistant humans (Kars et al. 2010). At 16 weeks, one to two rat offspring from individual litters were fasted for 15 h and the procedures described below were performed.

### Glucose tolerance test (GTT)

Glucose tolerance test was performed by standard methods as described (Chen and Nyomba 2003a,b; Dembele et al. 2006; Yao and Nyomba 2007, 2008; Yao et al. 2013). Glucose (30% w/v), 2 g/kg body weight, was intraperitoneally injected and saphenous vein blood (40 *μ*L) was sequentially collected for the determination of glucose (Ascensia Elite XL; Bayer HealthCare, Toronto, ON, Canada) and insulin (Ultrasensitive Rat Insulin Elisa kit; Crystal Chem, Downers Grove, IL). The intra‐assay coefficient of variation and the sensitivity of the insulin assay were 5% and 0.05 ng/mL, respectively. The area under the glucose or insulin curve (AUC) was computed by the trapezoidal rule, and the glucose‐insulin index was calculated as the product AUC_glucose_ × AUC_insulin_, which increases with insulin resistance (Cortez et al. 1991).

### Insulin tolerance test (ITT)

Insulin tolerance test was performed as described (Chen and Nyomba 2003a,b; Dembele et al. 2006; Yao and Nyomba 2007, 2008; Yao et al. 2013). Human recombinant insulin (Humulin R; Eli Lilly, Elkhart, IN), 0.75 IU/kg body weight, was intraperitoneally injected and saphenous vein blood was collected before and at 15, 30, 60, and 120 min, and used to determine glucose (Ascensia Elite XL; Bayer HealthCare). Insulin sensitivity was estimated during ITT by the first‐order rate constant of glucose disappearance (*K*_ITT_) computed as the slope of the regression line of the logarithm of blood glucose against time during the first 30 min.

### Insulin stimulation of Akt phosphorylation and glut4 translocation

The hindlimb muscles were exposed by carefully cutting through the skin. Regular insulin (Humulin R, 2 U/kg; Eli Lilly, Indianapolis, IN) or saline was injected into the left femoral vein followed by a rapid dissection of the gastrocnemius muscle 5 min later. This time frame is sufficient for activation of insulin signaling in rats (Chen et al. 2005). The muscle was trimmed of any visible fat, frozen in liquid nitrogen, and stored at −80°C until used.

### Muscle homogenate

Gastrocnemius muscle tissue (1 g) was homogenized on ice in a 20 mmol/L Tris buffer, pH 7.4, containing 140 mmol/L NaCl, 1 mmol/L CaCl_2_, 1 mmol/L MgCl_2_, 1% Triton X‐100, 10 mmol/L sodium pyrophosphate, 10 mmol/L NaF, 2 mmol/L Na_3_VO_4_, 2 mg/mL benzamidine, 50 *μ*mol/L trichostatin‐A, 10 mmol/L sodium butyrate, 1 mmol/L phenylmethylsulfonyl fluoride, and a protease inhibitors cocktail. Muscle homogenates were centrifuged for 10 min at 14,000 g and the supernatant was used for the measurement of Akt, phospho‐Akt, PTEN, and tribbles‐3.

### Muscle cell membranes

Muscle tissue cytosol and cell membranes were prepared by serial centrifugations as described (Chen et al. 2005). Gastrocnemius muscle tissue (1 g) was homogenized in 20 mmol/L Tris‐HCl buffer (pH 7.4) containing 0.25 mol/L sucrose, 1 mmol/L EDTA, 1 mmol/L phenylmethylsulfonyl fluoride, 0.01 mmol/L leupeptin, 50 *μ*mol/L trichostatin‐A, 10 mmol/L sodium butyrate, and 5 *μ*g/mL aprotinin. The homogenate was spun at 3000 g for 10 min at 4°C, and the supernatant was submitted to centrifugation at 100,000 g for 90 min. The new supernatant (cytosol) was collected and the precipitate (membranes) was resuspended in ice‐cold buffer by needle shearing. The cytosol and membranes were used for glut4 determination by western blot.

### Muscle nuclear extracts

Nuclei were isolated from skeletal muscle as described (Zahradka et al. 1989). Gastrocnemius muscle tissue (1 g) was homogenized in 10 mmol/L Hepes buffer (pH 7.5) containing 0.32 mol/L sucrose, 5 mmol/L KCl, 10 mmol/L MgCl, 5 mmol/L 2‐mercaptoethanol, 10 mmol/L NaF, 2 mmol/L Na_3_VO_4_, 2 mg/mL benzamidine, 1 mmol/L phenylmethylsulfonyl fluoride, 50 *μ*mol/L trichostatin‐A, 10 mmol/L sodium butyrate, and a protease inhibitors cocktail (lysis buffer). After filtration through 105 *μ*m mesh polypropylene membranes (Spectrum laboratories, Rancho Dominguez, CA) to remove large debris, the homogenates were spun at 1000 g for 8 min and the pellets were resuspended in lysis buffer containing 2.2 mol/L sucrose and centrifuged for 90 min at 80,000 g. The pellets were washed with the lysis buffer and spun for 10 min at 5000 g. The nuclear pellets were resuspended with a 20 mmol/L Hepes buffer (pH 7.9) containing 0.42 mol/L NaCl, 1 mmol/L EDTA, 2 mmol/L dithiothreitol, and 25% glycerol, and then centrifuged at 15,000 g for 30 min. The supernatants were dialyzed with Spectrum laboratories dialysis tubes (12–14 kDa cutoff) for 4 h in 20 mmol/L Hepes buffer (pH 7.9) containing 1 mmol/L dithiothreitol, 20% glycerol, 0.2 mmol/L EDTA, 50 mmol/L KC1 and 5 mmol/L MgCl_2_, and stored at −80°C until used for the determination of HDACs by western blot.

### C2C12 cell culture and treatment

C2C12 myoblasts were cultured at 37°C and 5% CO_2_ in Dulbecco's modified Eagle's medium (DMEM) supplemented with 15% fetal bovine serum (FBS), 100 IU/mL penicillin, and 100 *μ*g/mL streptomycin (growth medium). The cells were seeded on 6 well plates at 2 × 10^5^ cells/well and grown to 60% confluence in 2 mL of the DMEM. The cells were subsequently incubated for 24 h with ethanol or PBS before inducing differentiation. The appropriate ethanol concentration was selected by generating a dose–response curve of ethanol toxicity using MTT assay (Lee et al. 2010). MTT reduction decreased only by 10% at 80–120 nmol/L ethanol concentrations and by 16% at 140 nmol/L ethanol concentration. In subsequent experiments, we used 100 nmol/L ethanol, which is found in alcoholic humans (Lindblad and Olsson 1976) and is within the range of concentrations used in cell culture systems (Hong‐Brown et al. 2006; He et al. 2007). Cell differentiation was induced by incubation with DMEM containing 2% horse serum instead of FBS (differentiation medium) and supplemented with 1 mmol/L TUDCA (Hage Hassan et al. 2012), 100 *μ*mol/L CTPB (Balasubramanyam et al. 2003), 20 *μ*mol/L garcinol, 50 *μ*mol/L resveratrol, or 1 *μ*mol/L SAHA (Kadiyala et al. 2012). The incubation was continued for 5 days and the medium was changed daily.

### siRNA transfection

C2C12 cells were transfected with p300 siRNA according to the manufacturer's protocol. Briefly, the cells were seeded in antibiotic‐free normal growth medium supplemented with FBS and incubated at 37°C in a CO_2_ incubator until 60% confluency. The transfection was completed by incubating the cells for 6 h in p300 siRNA transfection medium supplied by the manufacturer without serum or antibiotics. Control cells were incubated with control (inactive) siRNA‐A. The cells were then washed and replaced in fresh normal growth medium. After 24 h, the cells were incubated in differentiation medium for 96 h.

### Akt and phospho‐Akt determination

Total Akt and phospho‐Akt were determined using Bio‐Plex Pro Cell Signaling Akt panels and Bio‐Plex 200 multiplex suspension array systems (Bio‐Rad).

### Western blotting

Western immunoblotting was performed as described (Yao and Nyomba 2007; Yao et al. 2013). Proteins (50 *μ*g/well), determined using the Bio‐Rad assay, were separated by SDS‐PAGE and electroblotted onto nitrocellulose membranes. The blots were blocked with 5% dry milk and incubated overnight at 4°C with the primary antibody at 1:1000 dilution. The blots were washed in Tris‐buffered saline‐Tween and then incubated with goat anti‐rabbit, donkey anti‐goat, or donkey anti‐mouse IgG‐HRP at 1:3000 dilution for 1 h at room temperature. Immune complexes were detected using the ECL chemiluminescent detection kit after exposing the blots to a Kodak X‐OMAT AR (XAR‐5) film. Protein contents were quantified by densitometry using NIH Image software, and the reading was normalized to *β*‐actin and expressed as fold of normal control.

### Real‐time PCR

Muscle mRNA expression of Glut4 and HDACs was determined by real‐time PCR using the Applied Biosystem 7500 thermocycler and reagents from InVitrogen (Carlsbad, CA) as described (Nguyen et al. 2012; Yao et al. 2013). First‐strand cDNAs were synthesized from total RNA using Moloney murine leukemia virus reverse transcriptase and oligo(deoxythymidine) primers. The reverse transcription product was amplified by real‐time PCR using specific primers (Table 1) and SYBR Green PCR master mix, with initial denaturation at 95°C for 10 min followed by 40 cycles of 15 sec at 95°C, and 1 min 30 sec at 60°C. Data were analyzed by the ΔΔCt method using ABI 7500 system software, and mRNA levels were normalized to *β*‐actin mRNA.

**Table 1.** Primer sequences.

Gene	Forward primer (5′–3′)	Reverse primer (5′–3′)
Glut4	ATCAACGCCCCACAGAAAGT	CCTGCCTACCCAGCCAAGT
HDAC1	CCATCAAAGGACATGCCAAGT	ACATTAGCATCGGCAAGTTGAA
HDAC3	CAGAGAGTCAGCCCCACCAA	CACCCACGTTGAAGGCATTAA
HDAC4	GGCAATTGCAGCCAAACTTC	GTGCACATCCCAGTCTACAATGA
HDAC5	CAAAAGCCCAGCGTCAATG	CACAGTCTCGGCCTCCTCTT
HDAC7	GGCTGGTCTATGACTCGGTGAT	GCGTGCTCGGGATGCTT
HDAC9	CCACCAGTCTGTGAATACGAATG	GAAACGGTCTCTGTCTCCTCTTG
ATF6	CCAGCAGAAAACCCGCATT	AGCCATCAGCTGAGAATTCGA
CHOP	TGGCACAGCTTGCTGAACAG	GTCAGGCGCTCGATTTCCT
XBP1	TGCGGAGGAAACTGAAAAACA	CATCCGGGCTTTCTTTCTATCTC
*β*‐actin	GCCAACCGTGAAAAGATGA	TTGCCGATAGTGATGACCTG

### Statistical analysis

Results are shown as the mean ± SE. Time‐dependent data (body weight, GTT, ITT) were analyzed by repeated measures (RM) analysis of variance (ANOVA; IBM SPSS, Chicago, IL). All the other data were analyzed by one‐way ANOVA. Tukey post hoc test was applied. Correlation coefficients are Pearson's product moments. *P* < 0.05 was considered significant.

## Results

### Body weight

**Figure 1.**
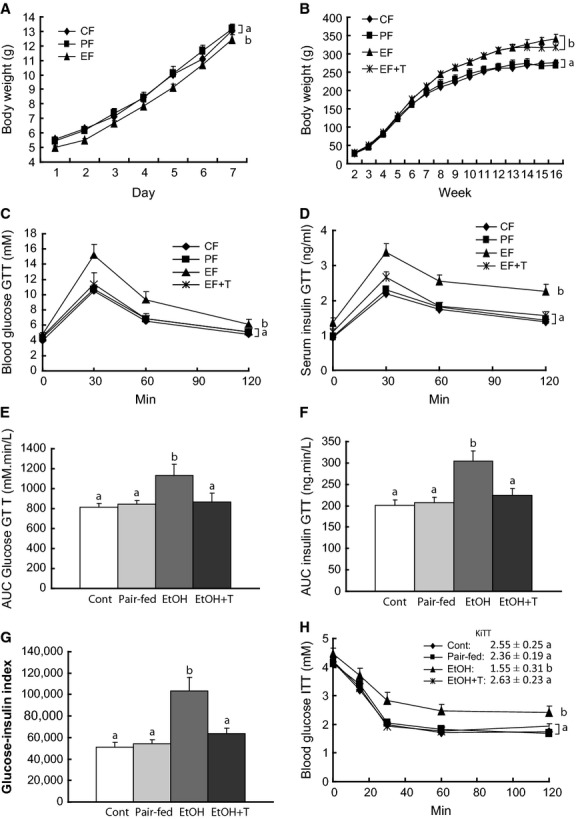
Effect of maternal ethanol on body weight, glucose tolerance, and insulin sensitivity in adult female rat offspring. Body weight during the first week (A) and from 2nd to 16th week (B) of age in offspring of dams exposed to ethanol (EF) versus control (CF) and pair‐fed (PF) dams. EF + T are EF offspring treated with tauroursodeoxycholic acid sodium. Blood glucose (C), insulin (D), area under the curve (AUC) for glucose (E), AUC insulin (F), and glucose‐insulin index (H) during glucose tolerance test (GTT). Blood glucose (G) during insulin tolerance test (ITT). *K*_ITT_ shown in the insert. Curves or means with different letters are significantly different (Tukey's test, *P* < 0.05).

Ethanol rats were significantly smaller than the other two groups during the first 7 days of life and then caught up, becoming bigger than the nonethanol groups as of the 8th week of age (Fig. 1). The weight gain in ethanol rats slightly slowed down with TUDCA treatment. There was no weight difference between pair‐fed and control groups.

### GTT and ITT

Glucose and insulin levels during GTT were all the time significantly higher in ethanol than in pair‐fed and control rats (Fig. 1). As a consequence, AUC glucose, AUC insulin, and the glucose‐insulin index were also greater in ethanol rats than in nonethanol rats. Treatment with TUDCA resulted in normalization of glucose and insulin levels as well as AUCs and the glucose‐insulin index during GTT in ethanol rats. During ITT (Fig. 1), the slope of the glucose disappearance curve was slower in ethanol rats than in the other two groups, which were similar. TUDCA treatment normalized the glucose disappearance curve in ethanol rats.

### Glut4 protein and mRNA in rat muscle

**Figure 2.**
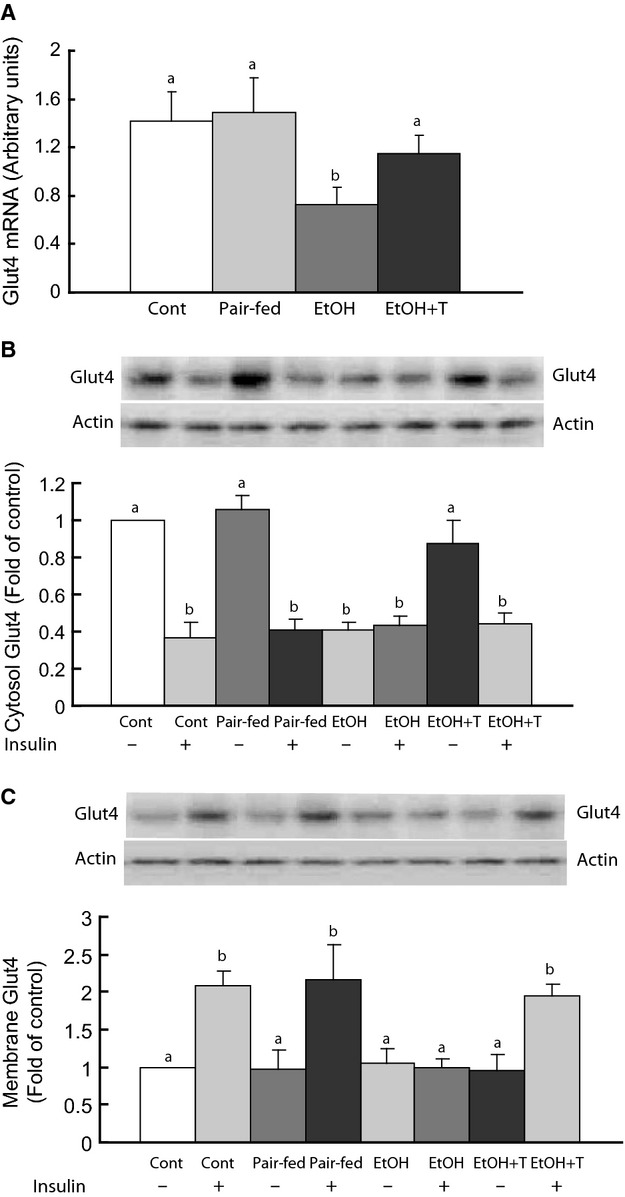
Glut4 mRNA (A) and glut4 protein in the cytosol (B) and membranes (C) of gastrocnemius muscle before and after insulin stimulation in adult rat offspring of dams exposed to ethanol (EtOH) versus control and pair‐fed dams. EtOH + T are EtOH offspring treated with tauroursodeoxycholic acid sodium. Means with different letters are significantly different (Tukey's test, *P* < 0.05).

We next examined glut4 protein and mRNA to explain changes in insulin sensitivity in ethanol rats and its improvement with TUDCA treatment (Fig. 2. In the basal state, glut4 mRNA and cytosolic glut4 protein levels were significantly reduced in ethanol rats versus nonethanol rats, and TUDCA treatment of ethanol rats normalized both glut4 mRNA and cytosolic glut4 protein levels (Fig. 2). At this time, before insulin administration, glut4 associated with the cell membrane showed similar levels among all groups. After insulin administration, glut4 in both cytosol and cell membrane remained unchanged versus basal in ethanol rats, whereas glut4 decreased significantly in the cytosol and increased in the membrane of control and pair‐fed rats. Treatment of ethanol rats with insulin in addition to TUDCA increased membrane glut4 content while depleting glut4 in the cytosol (Fig. 2).

### Phospho‐Akt and Akt inhibitors Pten and tribbles‐3 in rat muscle

**Figure 3.**
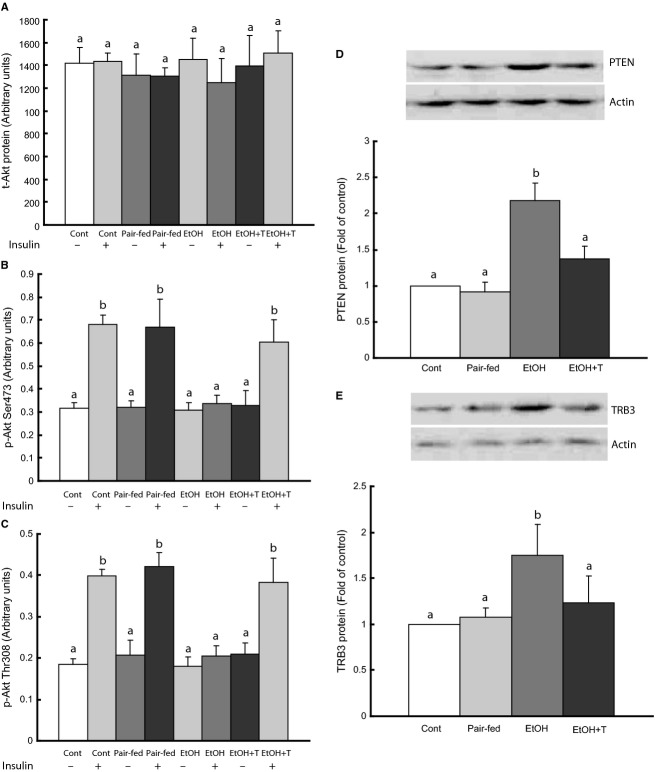
Akt activation in gastrocnemius muscle in adult rat offspring of dams exposed to ethanol (EtOH) versus control and pair‐fed dams. EtOH + T were EtOH offspring treated with tauroursodeoxycholic acid sodium. (A–C) Akt and p‐Akt before and after insulin stimulation. (D–E) PTEN and tribbles‐3 (TRB3) without insulin stimulation. Means with different letters are significantly different (Tukey's test, *P* < 0.05).

Because Akt phosphorylation plays an important role in glut4 translocation to the cell membrane, we determined phospho‐Akt levels before and after insulin stimulation in muscle homogenates (Fig. 3). Total Akt and basal phospho‐Akt levels were similar among rat groups. However, insulin‐induced Akt phosphorylation at both 473‐serine and 308‐threonine residues was impaired in ethanol rats compared with pair‐fed and control groups, and remained reduced after TUDCA treatment alone. When ethanol rats were treated with insulin in addition to TUDCA, Akt phosphorylation at both 473‐serine and 308‐threonine sites increased to the same level as controls. We next determined PTEN and tribbles‐3 content in muscle homogenates, because these proteins inhibit Akt activation by insulin and are increased by ethanol. Both PTEN and tribbles‐3 levels were increased in ethanol rats and were reduced to normal by TUDCA treatment.

### Histone deacetylases in rat muscle

**Figure 4.**
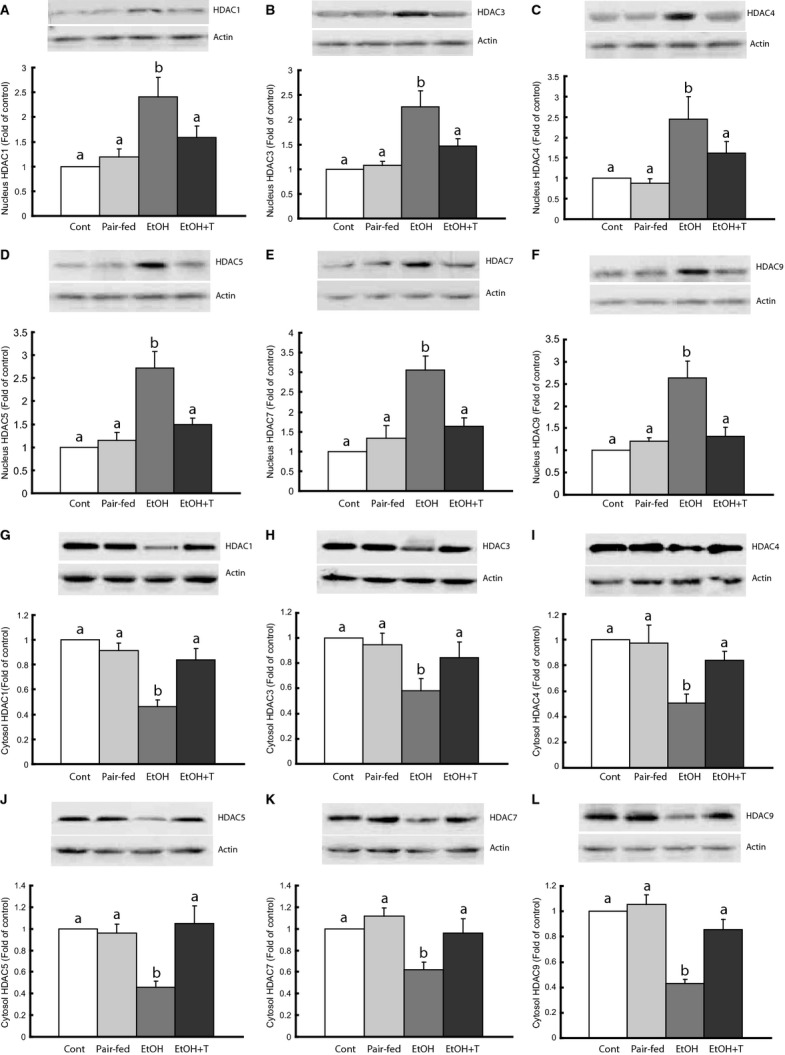
Class I and II histone deacetylase (HDAC) proteins in gastrocnemius muscle nucleus and cytosol in adult rat offspring of dams exposed to ethanol (EtOH) versus control and pair‐fed dams. EtOH + T were EtOH offspring treated with tauroursodeoxycholic acid sodium. Means with different letters are significantly different (Tukey's test, *P* < 0.05).

We tested whether class I and II HDACs are overexpressed in skeletal muscle of ethanol rats. The mRNA levels of these HDACs were not affected by ethanol or TUDCA treatment (not shown). However, the protein levels of these HDACs showed opposite changes in the nucleus and the cytoplasm. We found ~2.5‐fold increased protein content of HDACs of class I (HDAC1 and 3) and class II (HDAC4, 5, 7, and 9) in nuclear extracts of ethanol rats compared with control and pair‐fed groups (Fig. 4). TUDCA treatment reduced nuclear HDACs to levels similar to controls. In the cytosol, the HDACs were lower in ethanol rats than in offspring from control and pair‐fed dams, and were increased back to normal levels by TUDCA treatment.

### Correlation studies

Basal cytosolic glut4 content showed significant (*P* < 0.01) inverse correlations with nuclear HDAC levels that appeared to be of similar magnitude for all the HDACs (HDAC1: −0.730; HDAC3: −0.674; HDAC4: −0.679; HDAC5: −0.803; HDAC7: −0.794; HDAC9: −0.814). There were positive correlations between cytosolic glut4 and cytosolic HDAC levels that were of similar magnitude and significant (*P* < 0.01) for HDAC1 (0.677), HDAC4 (0.704), HDAC5 (0.837), HDAC7 (0.668), and HDAC9 (0.741), but not for HDAC3 (0.401). There was no correlation between HDACs and glut4 membrane content either before or after insulin stimulation.

### ER markers in rat muscle

**Figure 5.**
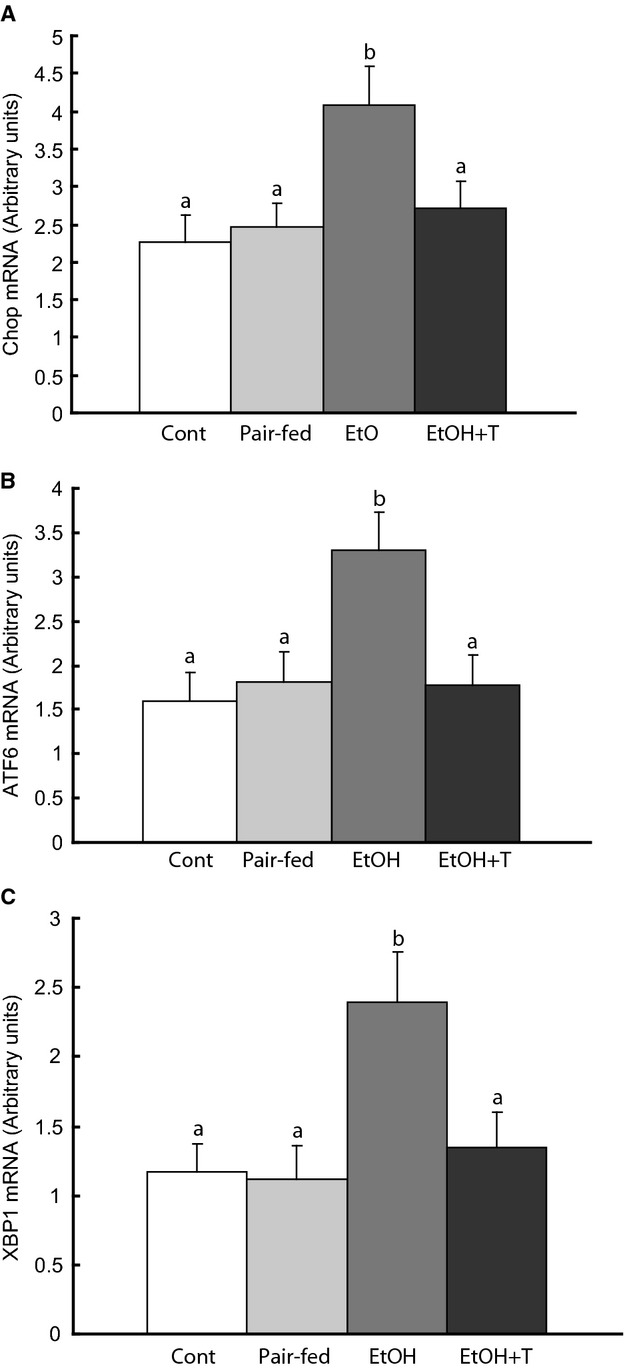
Endoplasmic reticulum (ER) stress markers in gastrocnemius muscle in adult rat offspring of dams exposed to ethanol (EtOH) versus control and pair‐fed dams. EtOH + T were EtOH offspring treated with tauroursodeoxycholic acid sodium. Means with different letters are significantly different (Tukey's test, *P* < 0.05).

The changes in the expression of tribbles‐3, a marker of ER stress, led us to investigate markers of unfolded protein response (UPR) in skeletal muscle in the four groups of rats by determining the expression of transcription factors representative of the three branches of the UPR, that is, IRE1, PERK, and ATF6. We found increased expression of XBP1, CHOP, and ATF6 in ethanol rats (Fig. 5). After treatment of these rats with TUDCA, however, these markers were comparable to those of control and pair‐fed rats.

### HDAC inhibition rescues glut4 expression in C2C12 cells

**Figure 6.**
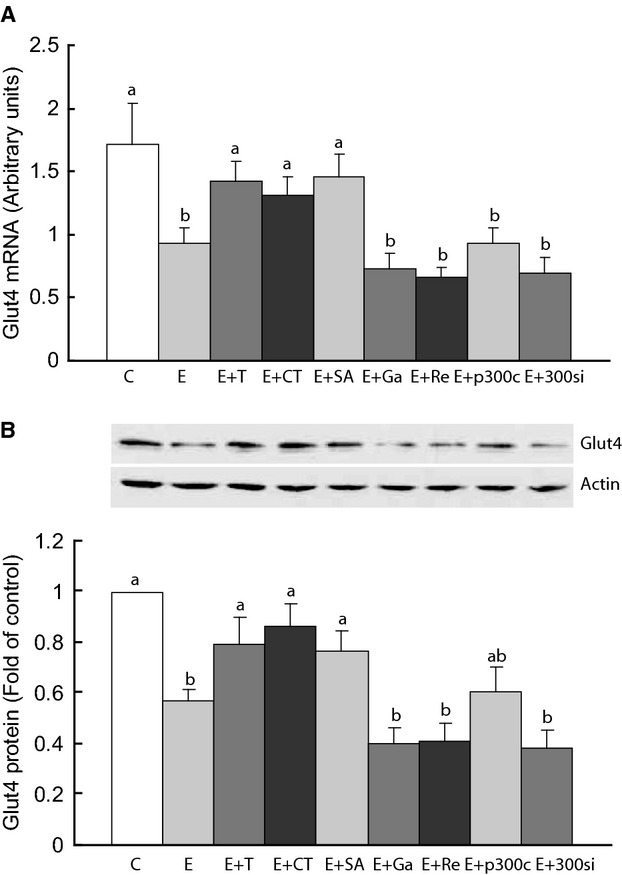
Glut4 mRNA and cytosol glut4 protein in C2C12 cells. The cells were untreated (control, C) or treated with ethanol (E), E plus tauroursodeoxycholic acid sodium (T), histone deacetylase (HDAC) inhibitor SAHA (SA), HDAC activator resveratrol (Re), p300 histone acetylase (HAT) activator CTPB (CT), or HAT inhibitor garcinol (Ga). HAT was also inhibited with p300 siRNA (si). p300c was a control solution for siRNA. Data shown as means ± SEM of 3 independent experiments carried out in triplicates. Means with different letters are significantly different (Tukey's test, *P* < 0.05).

To further investigate whether protein acetylation is instrumental in the effects of ethanol on glut4 in skeletal muscle, we treated C2C12 muscle cells with ethanol in association with modulators of acetylation. Ethanol reduced both glut4 mRNA and cytosolic level and this was reversed by TUDCA (Fig. 6). Inhibition of HDAC with SAHA and activation of p300 HAT with CTPB also reversed the effect of ethanol on glut4 mRNA and cytosolic level, whereas activation of HDAC with resveratrol or inhibition of HAT with garcinol or p300 siRNA had a nonsignificant additional effect on glut4 in ethanol treated cells (Fig. 6).

### HDAC inhibition does not affect unfolded protein response in C2C12 cells

**Figure 7.**
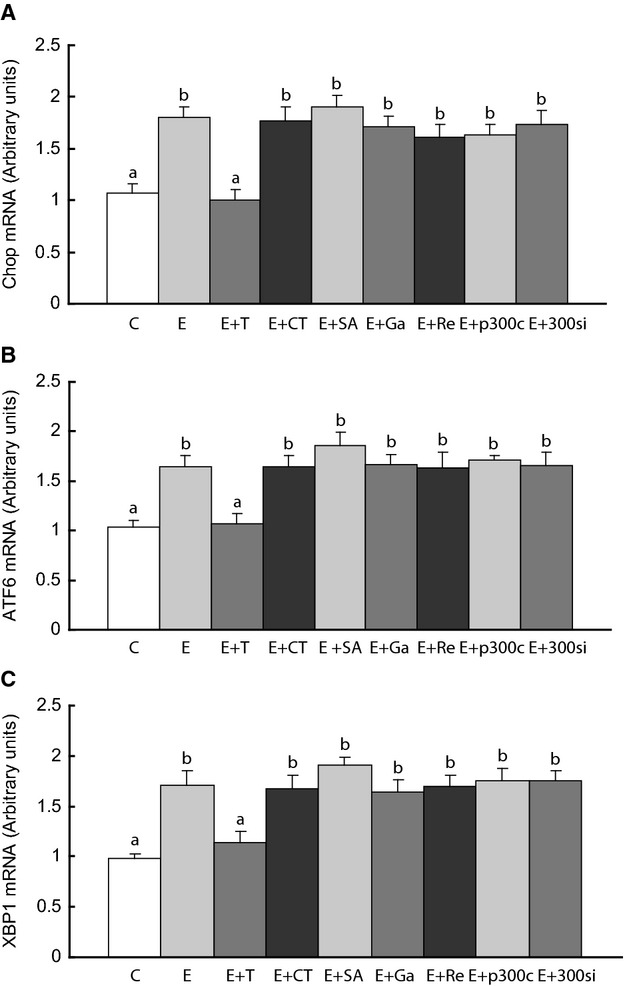
Endoplasmic reticulum (ER) stress markers in C2C12 cells. The cells were treated as stated in the legend of Figure 6. Means with different letters are significantly different (Tukey's test, *P* < 0.05).

The changes in UPR markers and the rescue of glut4 by HDAC inhibition in ethanol rat muscle led us to investigate the effect of ethanol and HDAC inhibition or HAT activation on ER markers in C2C12 cells. Ethanol exposure increased XBP1, CHOP, and ATF6 in these cells and TUDCA reversed the levels of these markers to normal (Fig. 7). However, inhibition of HDAC with SAHA or activation of p300 HAT with CTPB did not reverse the effect of ethanol on these markers. Similarly, inhibition of HAT with garcinol or p300 siRNA and activation of HDAC with resveratrol did not reverse the effect of ethanol on these UPR markers.

## Discussion

The present study demonstrates that adult rats exposed to prenatal ethanol have, not only a reduction of glut4 mRNA and protein in skeletal muscle but also impaired insulin stimulation of glut4 association with muscle plasma membrane. However, treatment with TUDCA normalized glut4 mRNA and protein and promoted glut4 translocation to the membrane in response to insulin, while TUDCA treatment alone had no effect on glut4 association with the cell membrane. Recent studies indicate that glut4 expression is regulated by the acetylation status of histones at the glut4 promoter. Histone acetylation by p300 HAT on the glut4 promoter enhances glut4 expression in skeletal muscle, whereas histone deacetylation by HDAC4 and 5 represses glut4 transcription in cultured muscle cells (Takigawa‐Imamura et al. 2003; Ojuka et al. 2012; Raichur et al. 2012) and adipocytes (Weems and Olson 2011). In one of these studies (Takigawa‐Imamura et al. 2003), chronic incubation of L6 myotubes with scriptide, a broad spectrum HDAC inhibitor and ER chaperone, increased glut4 cellular content and induced glut4 translocation, resulting in increased basal and insulin‐stimulated glucose uptake. However, glut4 mRNA level was unaltered and it was proposed that scriptide functions posttranslationally. In another report (Raichur et al. 2012), HDAC5 knockdown in human primary muscle cells or C2C12 myotubes increased glut4 expression and glucose uptake, while scriptide increased glut4 gene expression and glucose uptake; however, the effect of insulin on glut4 translocation was not investigated. An in vivo study of adult rats malnourished in utero showed impaired glut4 transcription secondary to deacetylation of the glut4 promoter by HDAC1 and 4 (Raychaudhuri et al. 2008). It appears, therefore, that both class I and II HDACs play a role in glut4 expression, and the increase in HDACs in the muscle nucleus of ethanol rats may explain the reduction of glut4 mRNA and protein expression, whereas inhibition of these HDACs by TUDCA has an opposite effect. These results agree with our previous report of reduced glut4 protein content in muscle of ethanol rats (Chen and Nyomba 2003a,b) and provide a mechanism of glucose intolerance in these rats, besides their increased levels of class I and II HDACs in liver nucleus (Yao et al. 2013). In that study, treatment with TUDCA also reduced hepatic nuclear HDACs and reduced gluconeogenic enzymes upregulated by these HDACs, with a reversal of glucose intolerance.

How metabolic signals control glut4 repression by HDACs is not fully understood. There is evidence that the repressive effect of class II HDACs on glut4 gene is relieved by certain protein kinases (PK) such as AMP‐activated PK (AMPK), calcium/calmodulin‐dependent PK2 (CaMK2), and possibly protein kinase D (PKD), which phosphorylate these HDACs leading to their nuclear egress, with as a result hyperacetylation of histones (McGee and Hargreaves 2010; Ojuka et al. 2012). Apparently, these PKs lead to hyperacetylation of histones in the vicinity of the myocyte enhancer factor‐2 domain, resulting in an increase of glut4 expression (McKinsey et al. 2001). On the other hand, cellular stress can increase nuclear HDACs and trigger histone deacetylation (Miura et al. 2008; Agudelo et al. 2011; Kahali et al. 2012; Zhang et al. 2014). As in the current study, we and others have shown that prenatal ethanol alters markers of UPR and increases oxidative stress in several organs (Dembele et al. 2006; Yao et al. 2013). Liu et al. (Liu et al. 2012) reported that both HDAC4 and 5 move out of muscle nuclei in response to reactive oxygen species production, which would support a nuclear egress of these HDACs during oxidative stress caused by ethanol. AMPK and PKD are both sensitive to cellular stress (McGee and Hargreaves 2010). In an apparent paradox, however, phosphorylation of class II HDACs by PKA results in HDAC retention in the nucleus (Ha et al. 2010; Chang et al. 2013). In addition, Liu and Schneider (Liu and Schneider 2013) have reported a net nuclear influx of HDAC4 after phosphorylation by PKA through *β*‐adrenergic stimulation, but a net nuclear efflux after phosphorylation by CaMK2. Their study, thus, showed opposing effects of HDAC phosphorylation depending on the phosphorylating pathway. In our study, HDAC levels increased in the nucleus and decreased in the cytoplasm of skeletal muscle after prenatal ethanol exposure. These mirror image levels without overall changes in HDAC gene expression agree with the notion that these HDACs shuttle to and from the cytoplasm. The data, therefore, suggest that the stress of prenatal ethanol may cause a net nuclear influx of HDACs, which is reversed by the antioxidant and ER chaperone TUDCA (Yao et al. 2013).

Histone acetylases and HDACs also regulate the acetylation status of cytosolic target proteins and their function. We have previously reported that prenatal ethanol increases PTEN and tribbles‐3 expression in skeletal muscle and liver, as confirmed in this study (Yao and Nyomba 2007, 2008). We have shown in the liver that PTEN and tribbles‐3 deacetylation by HDAC1 inactivates Akt, whereas acetylation of PTEN and tribbles‐3 by P300/CBP‐associated factor results in activation of Akt (Yao and Nyomba 2008), which in skeletal muscle are instrumental in glut4 translocation to the cell membrane in response to insulin. Thus, PTEN and tribbles‐3 prevent glut4 translocation in response to insulin by inhibiting Akt activation. PTEN and tribbles‐3 also play an important role in cellular stress and are affected by prenatal ethanol, which disturbs ER function and causes oxidative stress. Although TUDCA in this study increased glut4 expression in ethanol rats, TUDCA alone did not promote glut4 association with the cell membrane. In these animals, both insulin and TUDCA were required for an increase in glut4 translocation, suggesting that other insulin‐dependent mechanisms were required in addition to improved glut4 expression. One such mechanism is activation of Akt by insulin permitted by inhibition of PTEN and/or tribbles‐3. Okumura et al. (Okumura et al. 2006) have shown that PTEN is inhibited in the nucleus through acetylation by P300/CBP‐associated factor. Tribbles‐3 is also found in the nucleus (Hegedus et al. 2007) and we have previously found that tribbles‐3 is hypoacetylated and upregulated after prenatal alcohol exposure (Yao and Nyomba 2008). As nuclear, but not cytosolic, HDACs were increased by prenatal ethanol exposure, these results suggest that deacetylation of PTEN and tribbles‐3 likely took place in the nucleus. Deacetylation of these proteins would then trigger their translocation to the cytoplasm where they would prevent insulin‐induced Akt activation and glut4 translocation to the membrane. Conversely, TUDCA by reducing nuclear PTEN and tribbles‐3 levels through a reduction of nuclear HDACs would restore insulin response for Akt and glut4 translocation. It has also been reported that HDAC2 inhibits insulin response of the phosphoinositide 3‐kinase pathway in the liver through insulin receptor (IRS)‐1 deacetylation (Kaiser and James 2004). Although we did not study HDAC2 or IRS proteins, it is possible that restoration of IRS1 acetylation by TUDCA contributed to Akt phosphorylation and subsequent glut4 translocation in response to insulin.

In addition to in vivo studies, we investigated the effects of ethanol on glut4 in vitro. Ethanol exposure of C2C12 myoblasts had longlasting effects on differentiated myotubes, as glut4 expression was reduced and this was rescued by TUDCA and HDAC inhibition or HAT activation, but not by HDAC activation or HAT inhibition. The similarity of effect on glut4 between ethanol exposure and HDAC activation or HAT inhibition suggests that ethanol exposure before muscle differentiation programs muscle cells to express less glut4 likely via deacetylation of histones at the glut4 promoter. In contrast, the similarity between TUDCA effect and the effects of HDAC inhibitors or HAT activators supports the hypothesis that TUDCA acted on glut4 expression by inhibiting histone deacetylation or promoting histone acetylation at the glut4 promoter.

We also investigated whether the effect of HDACs on glut4 was related to ER markers. UPR markers were increased in ethanol exposed rats and C2C12 myotubes, but suppressed by TUDCA. However, HDAC inhibition or activation in C2C12 cells had no effects on UPR markers. Overall, the results in C2C12 cells indicate that ethanol and TUDCA have opposite effects on UPR and HDACs in both rats and C2C12 cells, but HDAC function influences glut4 expression independent of ER function, possibly via glut4 mRNA transcription rather than glut4 protein translation. The fact that TUDCA has an effect on distal UPR markers (ATF6, CHOP, XBP1) is consistent with the action of TUDCA as a chemical chaperone that stabilizes protein folding and reduces stress on the 3 ER branches. The lack of association between these distal UPR markers and the HDACs studied here suggests that these HDACs have no role in ER function.

**Figure 8.**
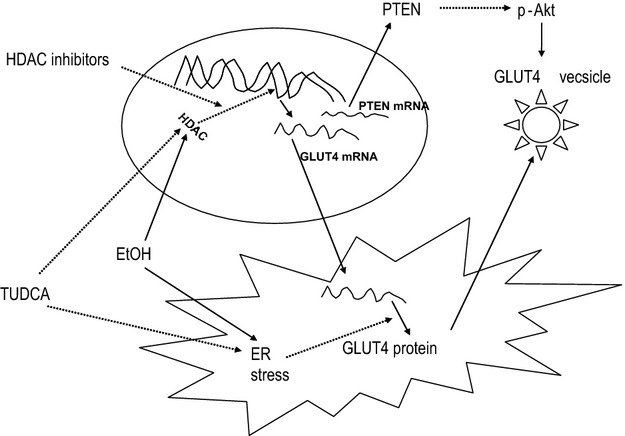
Ethanol (EtOH) increases nuclear localization of histone deacetylase (HDACs) and endoplasmic reticulum (ER) stress, which reduce glut4 transcription and synthesis, respectively. TUDCA blocks the effects of both HDACs and ER stress on glut4. HDAC inhibitors only block the effects of HDACs on glut4 transcription. Solid arrow indicates stimulation; dashed arrow indicates inhibition.

Thus, in ethanol rats, HDACs may cause insulin resistance of skeletal muscle both through a decrease of glut4 expression and through PTEN‐mediated Akt inhibition (Fig. 8). ER dysfunction caused by ethanol can also inhibit glut4 synthesis. To our knowledge, this is the first study of the effect of prenatal ethanol exposure on HDACs and glut4 regulation in skeletal muscle in vivo. The increase in nuclear HDACs and the resulting deacetylation of proteins may appear counterintuitive with views that ethanol metabolism, which results in acetate production, should increase acetylation of proteins. Although increased protein acetylation has been reported in response to acute ethanol exposure (Park et al. 2005; Pandey et al. 2008; D'Addario et al. 2011), the opposite is found with chronic ethanol exposure (Park et al. 2005; Pandey et al. 2008; Zhong et al. 2010; Guo et al. 2011). These findings support the possibility that prenatal ethanol exposure affects HDAC distribution not only in liver and skeletal muscle but also in multiple other organs.

A limitation of this study is that TUDCA was not administered to offspring not exposed to ethanol, and cultured control C2C12 cells were not treated with HDAC modulators. Therefore, the data could not be analyzed using a two‐way ANOVA as suggested by a reviewer. We used TUDCA exclusively in rats or cells exposed to ethanol in an attempt to correct the ER dysfunction prevalent after ethanol exposure. Drug treatment of control animals or cells although preferred is not a universal practice (Colell et al. 2001; Takada et al. 2012; Morton et al. 2013), and one‐way ANOVA has sometimes been used where a factorial analysis would have been better indicated (Morton et al. 2013; Vandoorne et al. 2013).

In conclusion, we demonstrate that ethanol exposure during pregnancy increases nuclear localization of class I and II HDACs and reduces glut4 expression and translocation to skeletal muscle membrane. HDACs also increase PTEN and tribbles‐3 levels and prevent Akt activation by insulin. These combined HDAC effects result in insulin resistance and glucose intolerance in rats exposed to ethanol. The results also indicate that HDAC inhibition by TUDCA restores glut4 expression and Akt activation by insulin, with improvement in insulin sensitivity and glucose tolerance.

## Conflict of Interest

The authors have nothing to disclose.
